# Parental knowledge, attitude, and practice on self-medication of antibiotics for children in bandung, indonesia: a questionnaire-based survey and module-based learning intervention

**DOI:** 10.1186/s12887-025-06084-8

**Published:** 2025-09-24

**Authors:** Dian Ayu Eka Pitaloka, Ariani Insyirah, Anisa Nabilah Oktariani, Cut Ainul Mardhiyyah, Nayla Majeda Alfarafisa

**Affiliations:** 1 Department of Pharmacology and Clinical Pharmacy, Faculty of Pharmacy, Universitas Padjadjaran, Sumedang, West Java Indonesia; 2 Center of Excellence in Higher Education for Pharmaceutical Care Innovation, Universitas Padjadjaran, Sumedang, Indonesia; 3 Faculty of Dentistry, Universitas Padjadjaran, Sumedang, Indonesia; 4 Faculty of Pharmacy, Universitas YPIB Majalengka, Cirebon, Indonesia; 5 Department of Biomedical Sciences, Faculty of Medicine, Universitas Padjadjaran, Sumedang, Indonesia

**Keywords:** Antibiotics, Children, Parents’ knowledge, Self-medication, Questionnaire

## Abstract

**Background:**

Parents’ role as primary decision-makers in medication administration is essential to ensuring proper antibiotic use. Despite its significance, only a few studies have delved into parental perspectives. Therefore, this study used a questionnaire-based survey and a module-based learning intervention to assess Indonesian parental knowledge, attitude, and practice related to antibiotic use in pediatric care.

**Methods:**

A total of 257 parents of children aged 0–11 attending postnatal healthcare centers in Arcamanik District, Bandung, Indonesia, participated in this study using a validated questionnaire. Additionally, we developed and implemented an education module using an on-site learning approach to assist a total of 57 parents who previously participated in the questionnaire-based study and agreed to participate in managing their children’s use of antibiotics. We also conducted pre- and post-tests to evaluate the improvement in knowledge.

**Results:**

The results showed that based on the questionnaire-based survey, about half of parents believed antibiotics were necessary for children’s illness (50%), and 64.5% considered their use essential when other treatments failed. Based on their previous symptoms, approximately 17.7% of parents misused antibiotics as stand-alone treatments. A significant 94.5% expressed the need for comprehensive information from healthcare providers regarding prudent use. The significant mean difference (45.79 ± 12.33; *P* = 0.00) between pre- and post-test analyses showed improvement in parental knowledge following the intervention.

**Conclusions:**

This study underscored a concerning lack of understanding among parents, leading to self-medication practices in Indonesia. Using the developed module, the educational intervention effectively increased parental awareness. This suggested the potential for tailored interventions to rectify misconceptions and promote responsible antibiotic use in pediatric care.

**Supplementary Information:**

The online version contains supplementary material available at 10.1186/s12887-025-06084-8.

## Introduction

Misconceptions about antibiotic use and resistance are prevalent among the general public [1]. The widespread use of antibiotics in communities is the main cause of resistance, which is the phenomenon where bacteria adapt to antibiotics and become resistant to their effects [2–4]. Among the demographic groups susceptible to colonization or infection by antibiotic-resistant bacteria, children are particularly vulnerable [5]. Over 20% of outpatient visits resulted in the prescription of antibiotics, according to statistical data [1]. This early and recurrent exposure contributes significantly to the emergence and perpetuation of antimicrobial resistance (AMR).

Pediatric patients in the United States continue to misuse and overuse antibiotics, thereby exacerbating the issue of resistance [6]. Furthermore, in Southeast Asia, including Indonesia, a significant concern arises, with 83% of children affected by *E. coli* strains that are resistant to commonly prescribed first-line antibiotics [6]. We cannot understate the essential role of parents as primary decision-makers in the acquisition and administration of medications in children, as they significantly influence the proper use of antibiotics in this population [7]. As stated by the World Health Organization (WHO), there is a significant knowledge gap regarding antibiotics and their appropriate use. For instance, a substantial 64% of respondents erroneously believed that antibiotics were effective against viral infections such as the common cold and flu. Although 72% of respondents acknowledged antibiotic resistance as a problem, a significant majority struggled to accurately define the concept [6, 8].

Various factors, including knowledge and beliefs, the severity of children’s illnesses, the availability of antibiotics, the children’s age, and the specific circumstances at hand, influence parents’ behavior regarding the use of antibiotics [7]. Studies investigating parental viewpoints on antibiotic use span a wide spectrum, from misconceptions to well-informed segments of the population. While current efforts primarily focus on healthcare providers to combat antibiotic resistance, understanding the perspectives of parents has received limited attention. It is important to identify these perspectives to comprehend the obstacles related to responsible antibiotic use, particularly for tailoring educational initiatives in specific communities [9]. The formulation of effective interventions aimed at parents necessitates a comprehensive grasp of not only knowledge regarding the appropriate indications for antibiotics, but also perceptions concerning the advantages and drawbacks of therapy. There is an urgent need to develop psychometric tools to assess the knowledge, attitude, and practice (KAP) of parents pertaining to this critical issue.

We created and validated the KAP questionnaire using structural equation modeling (SEM) analysis to assess Indonesian parental KAP regarding the use of antibiotics in pediatric care [10]. The validation of this standardized instrument is significant because it ensures the collection of accurate and well-defined data, enhancing the credibility of the results. Therefore, this study aimed to evaluate Indonesian parents’ KAP regarding self-medication for children using a validated questionnaire. The effectiveness of an education module for parents in increasing knowledge of antibiotic use was also assessed.

## Methods

### Study design and population

**Fig. 1:**
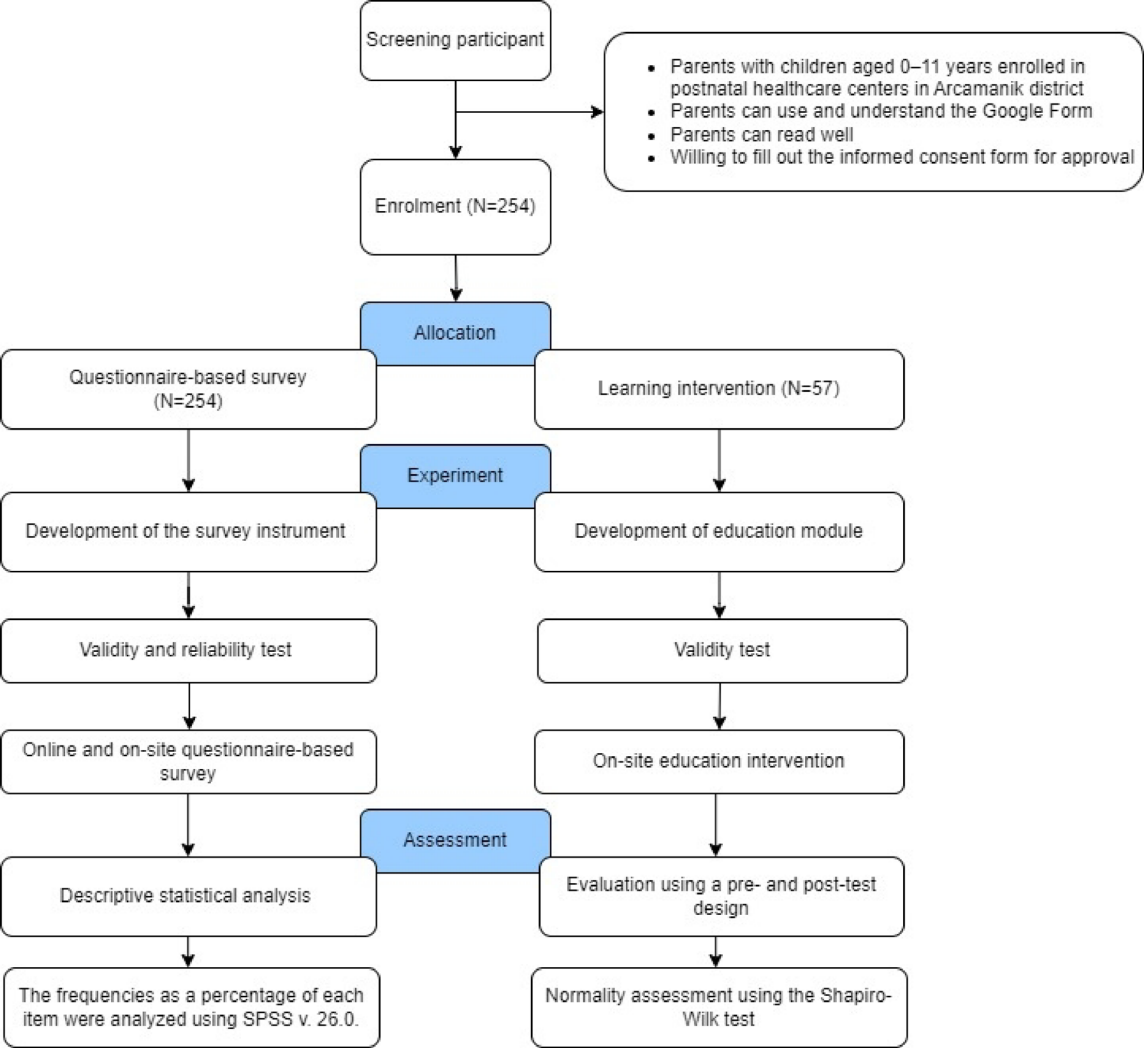
Flow chart of the study

Between January 23 and February 6, 2023, we conducted a cross-sectional study using both online and in-person interviews with 254 parents living in Arcamanik District, Bandung, Indonesia (Fig. 1). We targeted parents with children aged 0–11 years enrolled in the 17 postnatal healthcare centers as respondents. The inclusion criteria were to possess the ability to use and comprehend the Google Form, demonstrate proficient reading skills, and express a willingness to complete the informed consent form for approval. Furthermore, we invited all parents who participated in the survey to participate in a learning intervention about antibiotic use in children.

### Sample size calculation

We estimated the sample size of the study using Slovin’s formula. The Slovin formula is an ad hoc equation that provides an estimate of the sample size required to achieve statistically significant findings when sampling from a population with unknown characteristics [11].

$${\text{n}} = \:\frac{N}{{(1 + Ne^{2} )}}$$

where:

n: Sample size needed.

N: Population size.

e: Acceptable margin of error (0.05).

Given a total population of 2324 parents with children aged less than or equal to 11 years old, a minimum sample size of 138 was required for the questionnaire-based survey, with a margin of error of 5% and a confidence level of 95%. A total of 254 parents participated in this survey. In addition, for the module-based learning intervention with a population of 254 survey participants, 57 parents agreed to participate in the study.

### Ethical approval

Ethical approval was received from the Health Research Ethics Committee of Universitas Harapan Bangsa No. B.LPPM-UHB/1807/05/2023. Respondents provided written informed consent for participation. This study was conducted ethically in accordance with the World Medical Association Declaration of Helsinki.

### The questionnaire design

We developed the questionnaire in Bahasa Indonesia after reviewing related studies. These studies were selected based on a thorough examination of articles published in international journals from India [12], the United Arab Emirates [13], and Saudi Arabia [14], as well as adherence to government guidelines outlined in the Handbook of Antibiotic Use in Indonesia [15]. The questionnaire, which consisted of 47 items divided into five sections, included the first nine items as indicators of social demographics, while the remaining items addressed parents’ knowledge, attitude, practice, and parental facilitatory needs regarding antibiotic use in children using a Likert scale. We assigned scores ranging from 1 to 5, which represent “strongly disagree,” “disagree,” “doubtful,” “agree,” and “strongly agree,” to maintain clarity and avoid ambiguous intermediate values.

### Data collection and validity test

We conducted a two-week data collection process, administering a questionnaire through both online and on-site interviews. Parents in the four sub-districts of Arcamanik, Sukamiskin, Cisaranten Endah, Cisaranten Bina Harapan, and Cisaranten Kulon received the survey instrument through 17 postnatal healthcare centers using a convenience sampling. Subsequently, information gathered from respondents through Google Forms and paper-based questionnaires was extracted, coded, and entered into SPSS version 26.0 for analysis. Furthermore, we validated the questionnaire using SEM and performed statistical analyses using SPSS version 26.0 and AMOS version 26.9 [10]. The content validity for the scales was over 50%, and the reliabilities were above 0.6, respectively. We evaluated the model’s appropriateness and found the following parameter indicators: chi-square = 0.0004, CFI = 0.977, RMSEA = 0.044, CMIN/DF = 1.162, AGFI = 0.651, TLI = 0.973, and NFI = 0.860. The GFI parameter did not align with the output value of 0.718, but the convergent and divergent validity of scores indicated evidence in the expected direction [10].

### Development and implementation of educational modules

Next, we conducted online education for parents using a quasi-experimental design and a time series experiment [16]. We selected and placed parents as research subjects. In this study, we calculated the treatment observation using the difference in gain (T2–T1) following treatment. The study uses T1 as the pre-test (initial test) before treatment and T2 as the post-test (final test) after treatment. Parents receive an on-site learning intervention that utilizes module-based learning to address antibiotic use in children. A total of 57 parents who previously participated in the questionnaire-based study agreed to participate in this study, which included two on-site learning sessions on weekends as part of a four-week program in February 2023. Additionally, we facilitated an online discussion forum on weekdays to cover all topics related to the education module. The interval between the pre- and post-test was day 1 in the first week and day 28 in the fourth week of February 2023.

Based on the results of the questionnaire-based survey, we developed an educational module using the ADDIE (Analysis, Design, Development, Implementation, and Evaluation) model [17]. The survey generated four primary topics: (1) general knowledge of antibiotics; (2) the growing concern about antibiotic consumption and resistance in children; (3) common questions and answers related to antibiotic use in children; and (4) general guidelines and advice for parents regarding antibiotic use in children. To ensure convenient accessibility for parents, we created this module in both eBook and hard copy formats, assigning it an international standard book number (ISBN) of 978-623-8227-22-8 [18].

### Systematic evaluation of module implementation

**Fig. 2:**
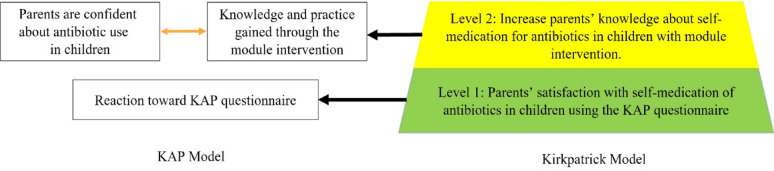
Theoretical framework of the study based on the KAP and Kirkpatrick models of evaluation

The Kirkpatrick model for evaluation of parental knowledge, attitude, and practice on self-medication of antibiotics for children guided us as we applied qualitative and quantitative methods as summative evaluations in the ADDIE process [19]. The four levels of evaluation are: (1) the parent’s response with self-medication of antibiotics in children using the KAP questionnaire; (2) the parent’s learning result and knowledge gained from the learning process with module intervention.; (3) the parent’s progress and change in behavior after using the knowledge on a daily basis; and (4) the impact that the parent’s performance has on the care given. For this report, we focus on the first two levels: level 1—the parent’s reaction to self-medication of antibiotics in children using the KAP questionnaire—and level 2— the parent’s learning result and knowledge gained from the learning process through pre-post module quizzes (Fig. 2). We asked parents to complete a pre-test before participating in the learning intervention and a post-test following the final meeting. The assessment of parent knowledge includes antibiotic use in children, as well as module-related concepts, skills, and tools within each module.

### Statistical analysis

We analyzed the response data using SPSS Statistics for Windows, Version 26.0. We generated graphic representations of the data using both MS Excel and MS Word to create informative graphs. We subjected the questionnaire responses to analysis using descriptive statistics, conveying the outcomes in terms of frequencies represented as percentages. We also scrutinized the normality of the data using the Shapiro-Wilk test. We performed a paired sample t-test to compare the mean knowledge scores before and after the test.

## Results

### Sociodemographics and participant characteristics

A total of 241 (94.9%) respondents were mothers, and 114 (44.9%) were in the age range of 31–40 (Table 1). More than 30% had a secondary education level, and approximately 95% had a non-medical profession with an average income per month (in IDR) of 0–1,500,000 (41.7%). A total of 184 (72.4%) had more than one child, with 34.3% having an average age in early childhood (3–5 years). Furthermore, more than half of the respondents were living in Cisaranten Kulon (50.4%), 17 (6.7%) were in Cisaranten Endah, 54 (21.3%) were in Cisaranten Bina Harapan, and 55 (21.7%) were in Sukamiskin.

**Table 1:** Demographics of respondents

Variable	Frequency, *n*/*N* (%)
Gender	
Male (father)	13/254 (5.1%)
Female (mother)	241/254 (94.9%)
Age	
21–30	92/254 (36.2%)
31–40	114/254 (44.9%)
41–50	29/254 (11.4%)
51–60	10/254 (3.9%)
61–70	7/254 (2.8%)
> 70	2/254 (0.8%)
Parents education level	
Illiterate	0/254 (0%)
Primary	7/254 (2.8%)
Middle	80/254 (31.5%)
Secondary	90/254 (35.4%)
Diploma	26/254 (10.2%)
University degree	48/254 (18.9%)
Postgraduate or above	3/254 (1.2%)
Parents profession	
Medical	12/254 (4.7%)
Non-medical	242/254 (95.3%)
Income/month (in million)	
0–1,500,000	106/254 (41.7%)
1,500,000–3,000,000	69/254 (27.2%)
3,000,000–4,500,000	45/254 (17.7%)
> 4,500,000	34/254 (13.4%)
Number of children	
1	70/254 (27.6%)
> 1	184/254 (72.4%)
Age of child	
Infancy: 0–24 months	80/254 (31.5%)
Toddler: 25–36 months	50/254 (19.7%)
Early childhood: 3–5 years	87/254 (34.3%)
Middle childhood: 6–10 years	60/254 (23.6%)
Late childhood: 11 years	30/254 (11.8%)
Child gender	
Male	136/254 (53.5%)
Female	148/254 (58.3%)
Residence	
Cisaranten Kulon	128/254 (50.4%)
Cisaranten Endah	17/254 (6.7%)
CIsaranten Bina Harapan	54/254 (21.3%)
Sukamiskin	55/254 (21.7%)
Homeless	0/254 (0%)

### General parental knowledge about antibiotics

**Fig. 3:**
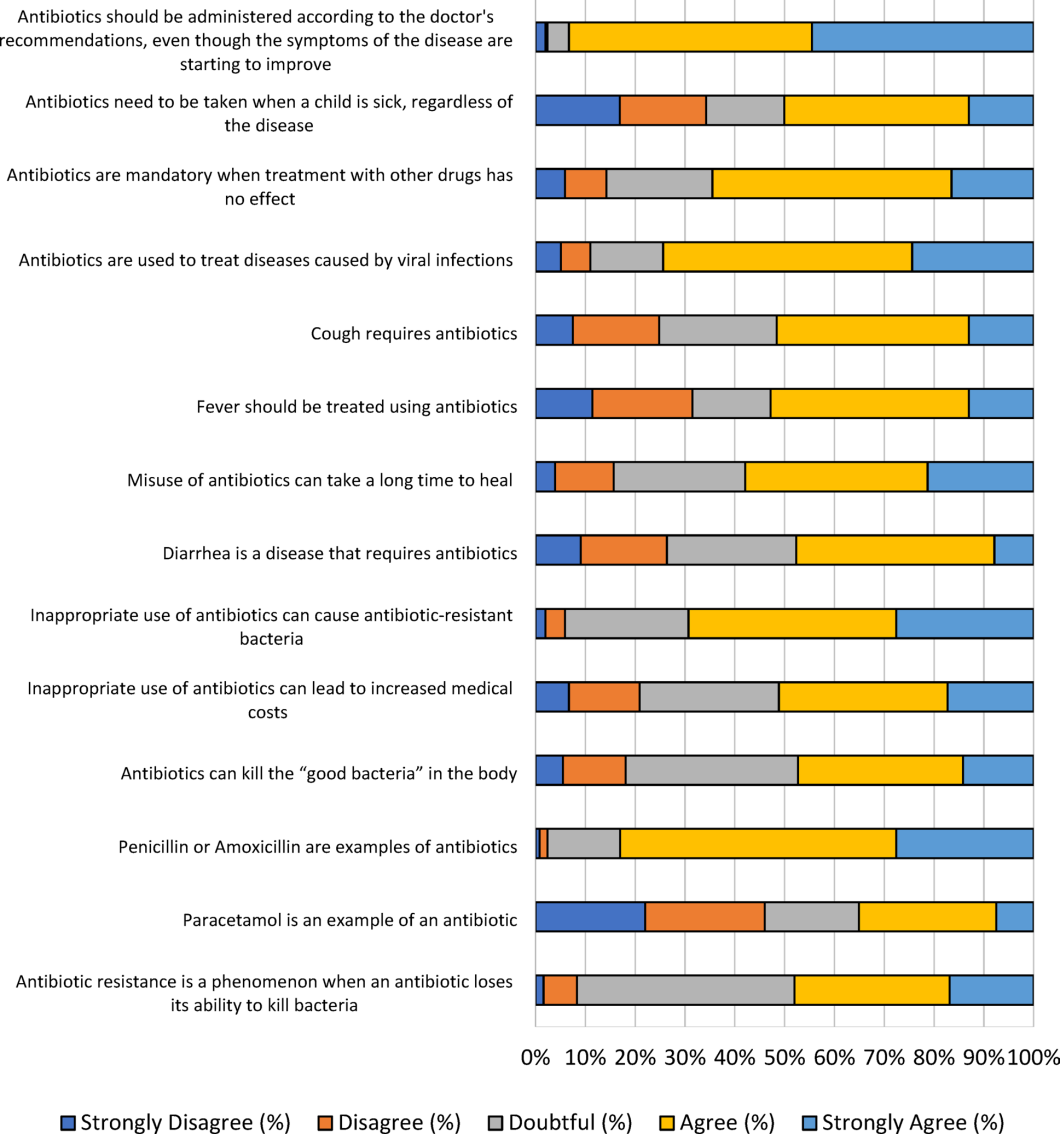
The score distribution of the “knowledge” dimension. A total 14 questions in likert scale were used to assess parental knowledge about antibiotics

Parental knowledge regarding antibiotic use in children varied widely. Approximately half of parents (93.3%) acknowledged that antibiotic use had to be based on the recommendations of doctors. Despite the fact that the disease’s symptoms were beginning to improve, the majority of parents still adhered to the doctor’s recommendations (Fig. 3). About 83.1% identified penicillin and amoxicillin as examples of antibiotics. A significant proportion of parents, namely 57.9%, were aware that inappropriate use of antibiotics would prolong the disease’s healing time due to the potential for antibiotic-resistant bacteria (69.3%) and increase medical costs (51.2%). Approximately half of parents (48%) recognized resistance as a phenomenon when antibiotics lose their ability to kill bacteria. However. Several parents believed that antibiotics were necessary for treating any illness in a child (50%), even when other treatments proved ineffective (64.5%). Based on the responses, antibiotics were considered necessary to treat viral infections (74.4%), Coughs (51.6%), and fevers (5%).

### Parental practice with using antibiotics in children

**Fig. 4:**
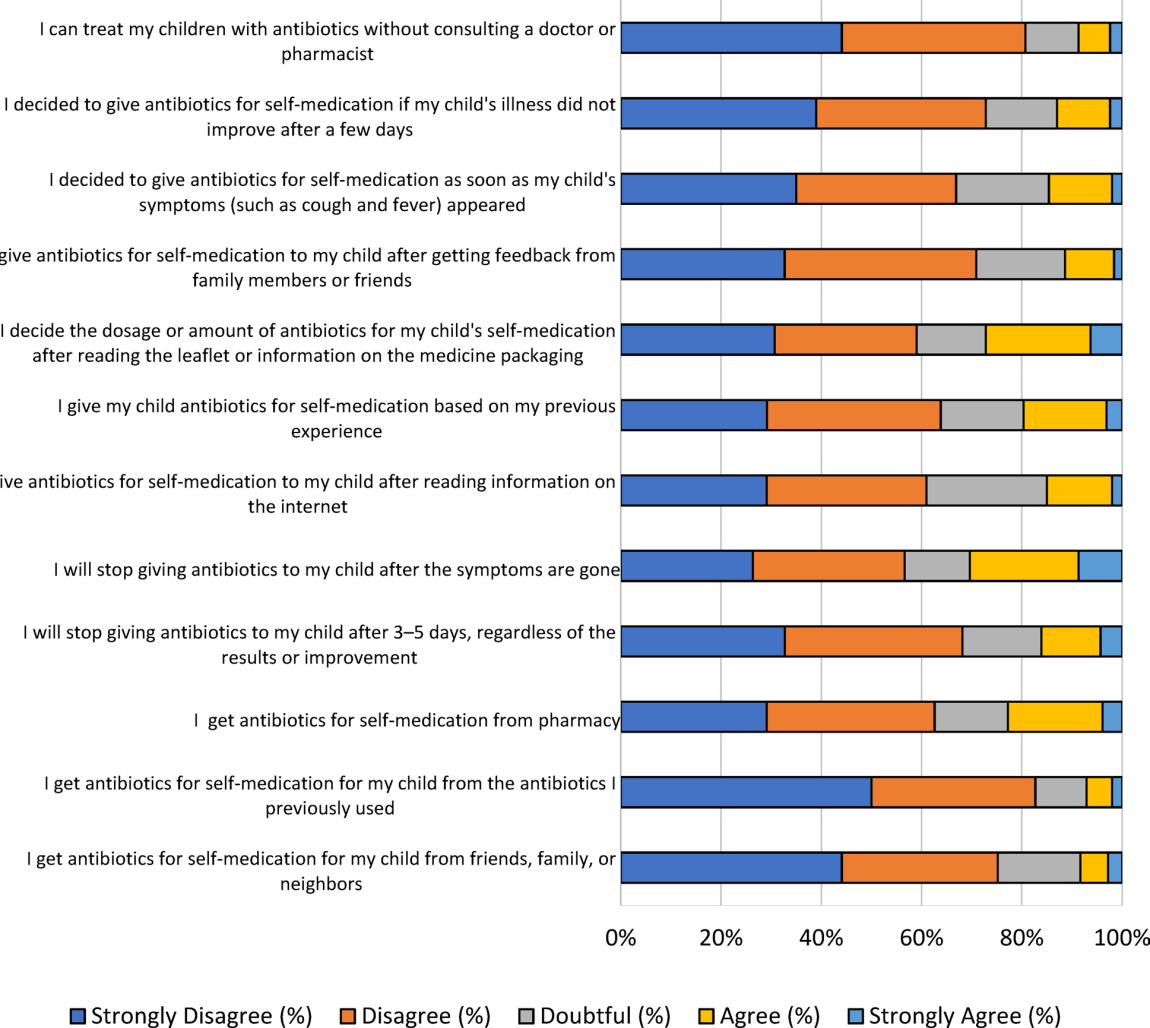
The score distribution of the “practice” dimension. A total 12 questions in likert scale were used to assess parental practice with antibiotic use in children

We further evaluated parental practice using antibiotics with children. The results showed that certain parents still did not understand how to use antibiotics properly for children. Some carried out self-medication without consulting a doctor or pharmacist (8.7%). However, the use of antibiotics by others was solely based on prior experience (19.6%), as illustrated in Fig. 4. Additionally, less than a quarter of parents purchased antibiotics for self-medication from pharmacies (22.8%), and a small percentage obtained antibiotics from friends, family, or neighbors (8.3%) after getting advice from family members or friends (11.4%). Parents initiated self-medication practice under various circumstances, such as when the illness did not improve after a few days (13%) at the onset of symptoms including cough and fever (14.6%). Alternatively, 15% of parents initiated self-medication after reading information on the internet. Individuals also determined the dose or amount of antibiotics after reading the leaflet or information on the medicine packaging (27.2%). Parents stopped giving antibiotics to children after symptoms disappeared (30.4%).

### Parental attitudes towards the use of antibiotics in children

**Fig. 5:**
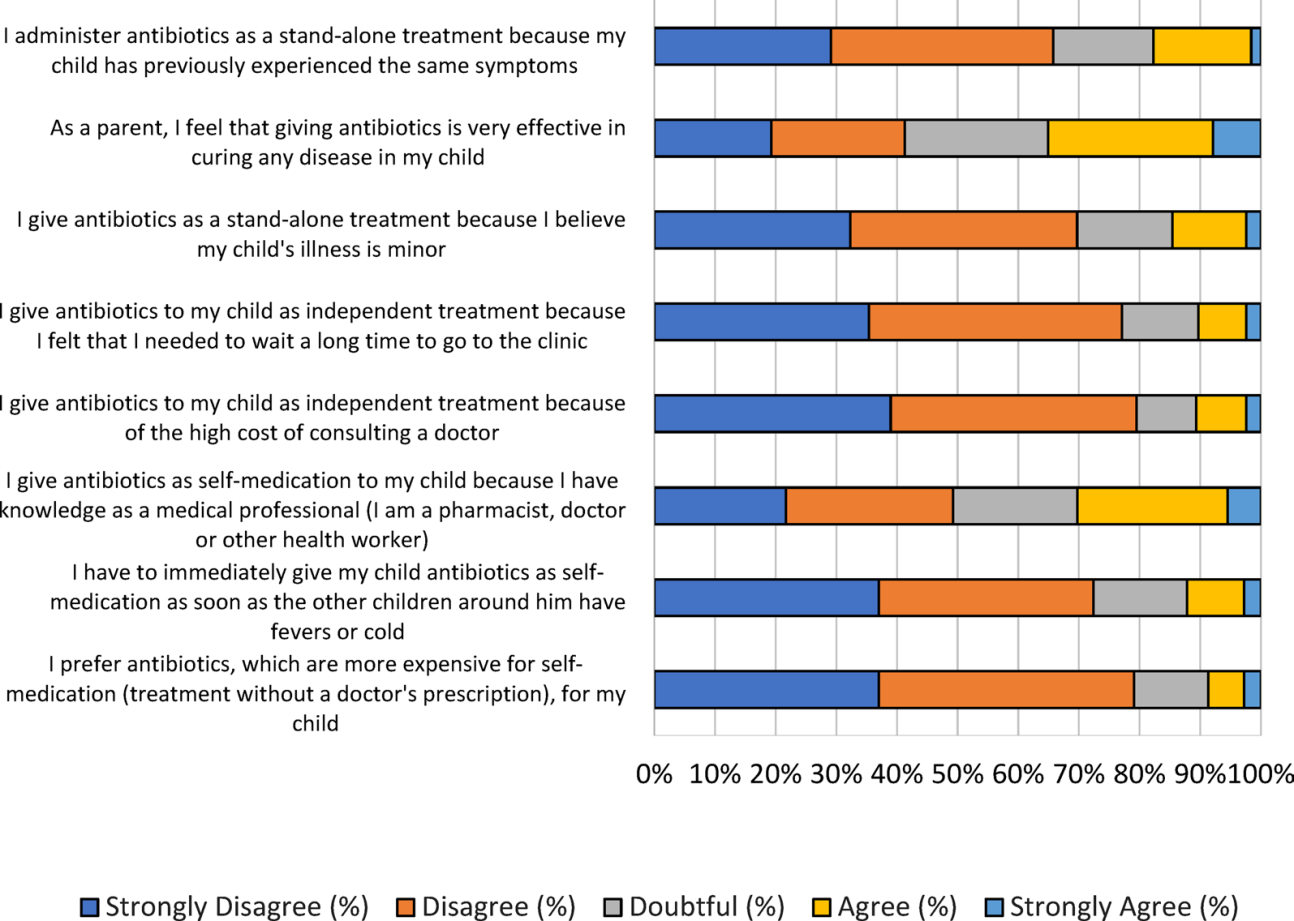
The score distribution of the “attitude” dimension. A total 8 questions in likert scale were used to assess parental attitude in antibiotics use in children

An evaluation of parental attitudes toward self-medication of antibiotics in children showed key insights. Approximately 17.7% of parents administered antibiotics as a stand-alone treatment because their children had previously experienced similar symptoms (Fig. 5). The severity of the illness was mild (14.6%), and due to its effectiveness in curing any diseases (35.1%), parents opted to self-medicate. Other factors influencing parental decisions to self-medicate include the perceived delay in obtaining a prescription from the clinic (10.3%), the urgency to administer antibiotics immediately when children have a fever or cold (12.2%), and the high cost associated with consulting a doctor (10.7%). About 30.3% of parents possess knowledge from medical professionals, including pharmacists, doctors, and other health workers. A small proportion (8.7%) prefer more expensive antibiotics for self-medication.

### Parental facilitatory needs in antibiotics use in children

**Fig. 6:**
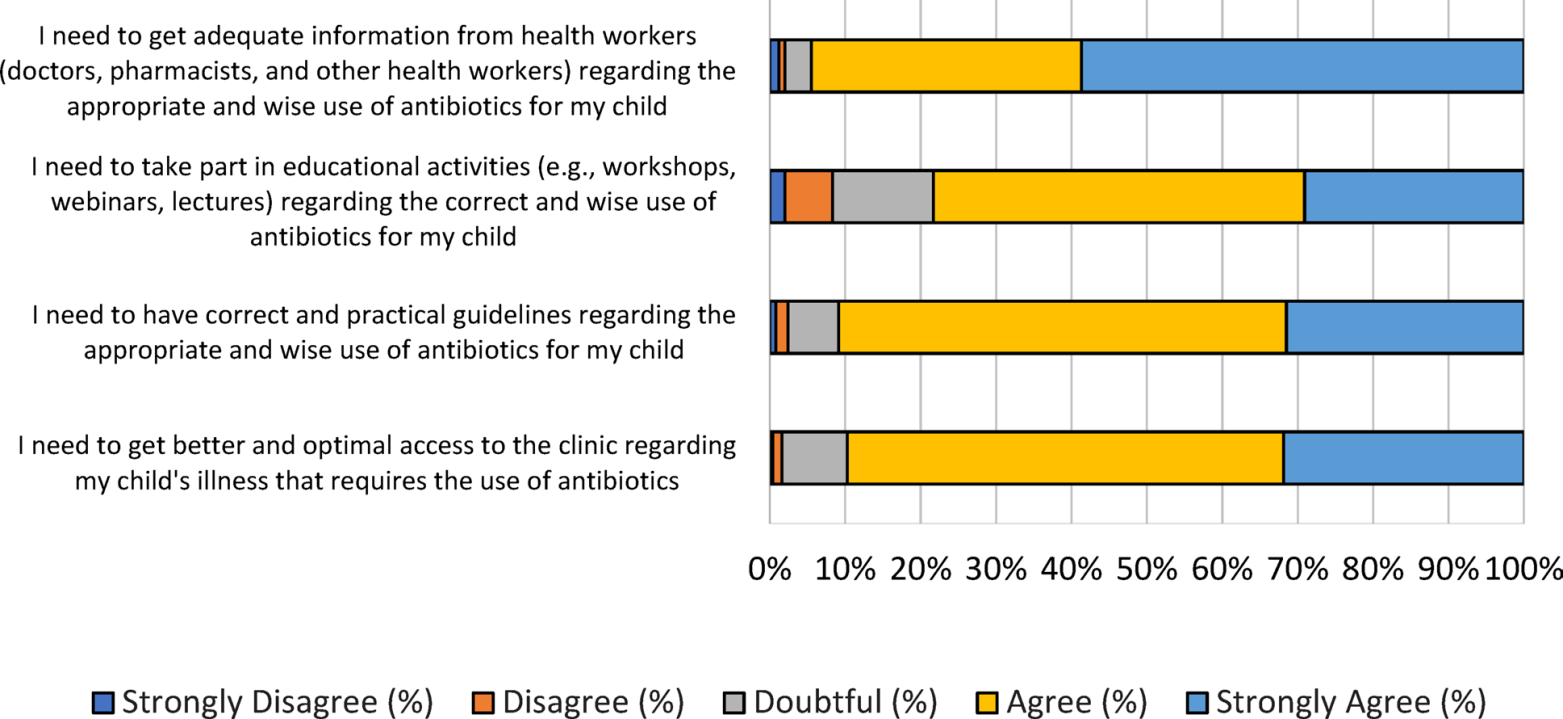
The score distribution of the “facilitatory needs” dimension. A total 4 questions in likert scale were used to assess parental facilitatory needs in children’s antibiotic use

We also evaluated the needs of parents to facilitate the use of antibiotics in children. Approximately 94.5% of parents expressed the necessity of obtaining adequate information from health workers, including doctors, pharmacists, and others, regarding the appropriate use of antibiotics for children (Fig. 6). More than half (78.3%) agreed to take part in educational activities such as workshops, webinars, and lectures. Approximately 90% expressed the need to have correct and practical guidelines regarding the appropriate use of antibiotics for children. For illnesses that necessitated antibiotic use, about 80% of parents needed better and optimal access to the clinic.

### Bivariate analysis

We performed bivariate analyses to determine the relationships between each question about knowledge, attitude, practice, and other variables specific to the participants. The bivariate analysis of general parental knowledge reveals a significant relationship between parents’ education level and other variables, likely due to their extensive knowledge, as demonstrated by their responses to most of the given questions. About 50% (7/14) of the questions were true. Furthermore, monthly income and residence provided approximately 35% (5/14) of the true answer, making it the second significant variable correlated to knowledge after parent education (supp.1).

In line with the previous result, a bivariate analysis of sociodemographic variables and parental attitudes toward antibiotic use in children found that monthly income had a significant relationship with attitude (*p* < 0.05), giving only 2 out of 8 questions a higher score (suppl. 2). The number of child variables also has the same relationship with attitude. Lastly, variable gender had a significant relationship with parental experience about antibiotic use in children (*p* < 0.005), with a female having a higher experience score compared to a man (suppl. 3). About 41% (5/12) of the questions had a higher score and a significant p value.

### Health education intervention impact

We conducted pre- and post-tests using a modular approach for parents of children aged 0–11 years to determine the success of the health education intervention. Based on the results, Four questions out of a total of 10 showed improvement of more than 50%. This improvement was evident in questions 1, 3, 5, and 9 (Table 2).

**Table 2:** The questions and result of pre-test and post-test for the health education intervention

No	Questions	Pre-Test (%)		Post-Test (%)		Right Answer	Progress (%)	NGain *P*-value
		Correct	Incorrect	Correct	Incorrect			
1	Antibiotic use must be stopped. Even though the symptoms are starting to improve, we must stop using antibiotics	36.84	63.16	87.72	12.28	False	50.88	0.81
2	Antibacterial resistance occurs when bacteria become resistant to antibiotics. making the treatment more difficult	56.14	43.86	94.74	5.26	True	38.60	0.88
3	A sore throat is an example of a disease in my child that requires antibiotics	31.58	68.42	85.96	14.04	False	54.38	0.79
4	If the disease’s symptoms have improved, you can stop taking antibiotics. This is a crucial step in preventing antibiotic resistance	31.58	68.42	73.68	26.32	False	42.10	0.62
5	My child has been experiencing green snot and coughing for the past four days. The symptoms, which are accompanied by a fever of up to 38 °C, indicate that my child is suffering from sinusitis	10.53	89.47	63.16	36.84	True	52.63	0.59
6	I should get a strep test but do not need antibiotics unless the test and culture results are positive if my child has a sore throat with a fever and the doctor says her throat is very red and has pus. However, the doctor has not detected any other serious health issues	28.07	71.93	75.43	24.57	True	47.36	0.66
7	If my child has a cold, I will give her antibiotics to prevent ear infections and sinusitis	52.63	47.37	68.42	31.58	False	15.79	0.33
8	My child may only need pain relievers (ibuprofen, paracetamol) and rest when the doctor tells me that my child has an ear infection	19.29	80.71	64.91	35.09	True	45.62	0.57
9	Abdominal pain, such as nausea and vomiting, diarrhea and shortness of breath are signs that my child is experiencing side effects (adverse and unwanted effects) from taking antibiotics	8.77	91.23	70.17	29.83	True	61.4	0.67
10	Antibiotics are medications commonly used to treat bacterial infections	45.61	54.39	94.73	5.27	True	49.12	0.90

Respondents’ responses to question number 9 about abdominal pain, which includes nausea, vomiting, diarrhea, and shortness of breath as examples of unwanted side effects from taking antibiotics, showed the greatest improvement. In the pre-test, only 8.77% of respondents correctly answered the question, compared to 70.17% in the post-test. There was a 61.4% improvement in answering question number 9. Furthermore, 31.58% of respondents were aware that sore throat was an example of a disease in children requiring antibiotics in question number 3 for the pre-test. This value increased from 54.38 to 85.96% in the post-test, indicating increased knowledge among respondents.

Based on the results in question number 5, a total 89.47% of parents were not aware that green snot and coughing for 4 days accompanied by a fever of up to 38◦C were symptoms of an illness, indicating the potential occurrence of sinusitis. However, education led to an improvement, as only 36.84% of parents were unaware of the given question, demonstrating a 52.63% improvement. When the symptoms gradually improve, only 36.84% correctly answered question number 1 about antibiotic use. Post-test results showed that 87.72% could answer the question, representing a 50.88% improvement in knowledge.

We also conducted a paired t-test analysis on the pre-test and post-test scores. The results showed that the mean obtained before and after the health education intervention was 32.10 ± 16.31 and 77.89 ± 11.96, respectively. A significance score of P 0.000 (*P* < 0.05) indicated an increase in the significant mean difference between before and after the health education intervention, estimated at 45.79 ± 12.33 with a confidence interval value of 36.96–54.61.

The variable transformation test in the NGain test yielded a mean result of 0.68, indicating a level of improvement both before and after the intervention. The results indicated that following a health education intervention, improvements in KAP values ranged from 0.3 to 0.7, falling in the medium range but approaching a high value when the score was 0.7 or above.

## Discussion

Parental KAP plays a crucial role in determining the suitability of antibiotics for children. This is the first study to investigate the characteristics and psychometrics of parental KAP in Bandung in relation to children’s self-medication of antibiotics. To accomplish the objective, we developed a KAP questionnaire. We repeated the reliability tests to confirm the validity and reliability of the questionnaire [10].

Self-medication with antibiotics for children is prevalent among their parents. According to reports, 10% of parents in Yunnan and 60% in Mongolia treated children with antibiotics. In rural China, the level of self-medication with antibiotics among children during a year reached 62% [20]. Nepal et al. (2023) conducted a study about the effect of an educational intervention targeted at parents of young children on non-prescribed antibiotic consumption in Nepal. The results showed that 35.1% of parents administered antibiotics as treatment for children [21]. Some parents keep antibiotics at home because they are leftovers from previous uses [21]. This study obtained a similar result, as 17.7% of parents used the leftover antibiotics when the children experienced the same symptoms. This practice perpetuates misunderstandings about antibiotics, as it frequently takes place without a doctor’s prior examination and diagnosis to determine whether the illness stems from an infection or if a culture test is required.

According to a Greek study, 87% respondents agree that the emergence of antimicrobial resistance was contributed by inappropriate prescriptions of antibitiotics [22]. More than half of the respondents requested antibiotics. Parents also intended to save antibiotics at home [23] and practice self-administration [24]. This is in line with the study conducted by Wun et al. (2012), which parents resorted to self-medicating children with antibiotics (saving them at home and using leftovers from the last prescription) due to concerns about long wait times at clinics and high consultation costs [25]. Practicing self-medication with antibiotics poses risks, as children might not complete the regimen, leading to an incomplete recovery. Parents’ desire for antibiotics for their children was primarily driven by fever, despite their concerns about potential side effects, complicating the situation [25].

Our health education intervention aimed to educate parents about the use of antibiotics in their children, based on the observation that most parents lack adequate information and knowledge from healthcare professionals, including pharmacists (Fig. 5). This intervention has improved parental awareness regarding antibiotic misuse in their children. Our findings suggest that parents’ educational level and family income influence their decision-making regarding their children’s health based on bivariate analysis (suppl. 1). Hahn et al. (2015) conducted an empirical study and found that basic educational expertise and skills, such as fundamental knowledge, reasoning ability, emotional self-regulation, and interactional abilities, are critical components of health. It is also crucial to implement programs that promote health equity between low-income and educational outcomes [26].

The Indonesian government, through Minister of Health Regulation Number 28 of 2021, stated that antibiotic use must be based on doctors’ prescriptions. Pharmacists are responsible for ensuring the continued supply and accessibility of antibiotics, promoting health policies, and monitoring them, as well as educating patients on antibiotic use [27]. Antibiotics should be used with caution. This is necessary to combat the growing trend of bacterial resistance [28]. In this study, more than 90% of parents expressed the need to have correct and practical guidelines regarding the appropriate use of antibiotics for children.

This study offers several strengths. First, this study is the first in Indonesia to introduce the concept of measuring the level of parental KAP in children who self-medicate with antibiotics. We also validated the framework for questionnaire development. We employed SEM analysis to construct studies. It is still possible to continuously evaluate and integrate this questionnaire into intervention strategies.

### Limitation

This study’s limitations include the use of convenience sampling, which poses a potential risk of retrieval bias in the sample. To address this drawback, we should take samples from a broader population across districts or cities in Indonesia. Despite meeting the study’s minimum sample size requirement, respondents were younger in the range of 21–40 years (92%), non-medical personnel (95.3%), and some had secondary education (66.6%). Expanding the samples to include a broader range of ages, including medical professionals and individuals with higher education levels, would enhance better representation and validity. In addition, the significant difference between father and mother respondents may introduce bias regarding the relationship between gender and parental experience with antibiotic use in children, so equal proportions may be needed for future studies. This is to prevent bias towards areas other than the districts or cities targeted for the study. Selection bias could also occur, as parents with a stronger interest in the independent use of antibiotics may be more inclined to respond and participate in the survey.

### Implication

In order to ensure effective antibiotic use, parents, as the major decision-makers in medicine administration, are vital. Few studies have examined the perspectives of parents. Despite the significance of this issue, our study provides the design and psychometric properties that make it an effective and useful tool. Larger respondents have extensive educational strategies. Our result also implies that parental educational attainment and family income serve as a foundation for parents’ decision-making regarding their children’s health. Future evaluations should take into account our study’s findings. This study provides a strong foundation for this type of analysis.

## Conclusion

In conclusion, this study delved into the prevailing misconceptions and challenges surrounding antibiotic use and resistance, with a specific focus on the vulnerable demographic of children. The results highlight troubling trends, including a significant number of parents who believe that antibiotics are essential for any child’s illness. Additionally, there are gaps in their understanding of proper use, which can lead to self-medication with leftover antibiotics. The educational intervention, incorporating the developed module, demonstrated outstanding effectiveness in enhancing parental awareness. Furthermore, the statistically significant improvement in post-test scores compared to the pre-test suggests that targeted health education has a positive impact. This underscores the potential for tailored interventions to rectify misconceptions and promote responsible antibiotic use. Targeted educational initiatives, informed by the specific beliefs and practices identified in this study, are crucial in promoting responsible antibiotic use. By empowering parents with accurate information, healthcare professionals can play a critical role in safeguarding antibiotic efficacy and ensuring the well-being of children in the face of resistance challenges.

## Supplementary Information

Below is the link to the electronic supplementary material.

Supplementary Material 1

## Data Availability

The datasets used and/or analyzed during the current study are available from the corresponding author on reasonable request.

## Declarations

### Clinical trial number

Clinical trial number is not required in this study because it does not include biomedical interventions, including new treatments (such as new vaccines, drugs, food choices, dietary supplements, and medical devices) and known interventions that require further study and comparison.
