# Supplementary material for: Parental knowledge, attitude, and practice on self-medication of antibiotics for children in bandung, indonesia: a questionnaire-based survey and module-based learning intervention
Source: BMC Pediatr. 2025 Sep 24;25:687. doi: 10.1186/s12887-025-06084-8 (PMC12462208; doi:10.1186/s12887-025-06084-8)
Supplement: Supplementary file 1 — Supplementary Material 1 [file 12887_2025_6084_MOESM1_ESM.docx]

Parental knowledge, attitude, and practice on self-medication of antibiotics for children in Bandung, Indonesia: A questionnaire-based survey and module-based learning intervention

Dian Ayu Eka Pitaloka^1,2^, Ariani Insyirah^1^, Anisa Nabilah Oktariani^3^, Cut Ainul Mardhiyyah^1,4^, Nayla Majeda Alfarafisa^5^

1. Department of Pharmacology and Clinical Pharmacy, Faculty of Pharmacy, Universitas Padjadjaran, Sumedang, Indonesia
2. Center of Excellence in Higher Education for Pharmaceutical Care Innovation, Universitas Padjadjaran, Sumedang, Indonesia
3. Faculty of Dentistry, Universitas Padjadjaran, Sumedang, Indonesia
4. Faculty of Pharmacy, Universitas YPIB Majalengka, Cirebon, Indonesia
5. Department of Biomedical Sciences, Faculty of Medicine, Universitas Padjadjaran, Sumedang, Indonesia

Correspondance:

Dian Ayu Eka Pitaloka

Address: Department of Pharmacology and Clinical Pharmacy, Faculty of Pharmacy, Universitas Padjadjaran, Jl. Raya Bandung-Sumedang KM 21, Sumedang, West Java, 45363, Indonesia.

Telephone/Fax: (022) 7796200/ (022) 7796200

e-mail address: dian.pitaloka@unpad.ac.id

**Supplementary 1.** Bivariate analysis of general parental knowledge about antibiotics

| **Variable** | **Q1** | **Q2** | **Q3** | **Q4** | **Q5** | **Q6** | **Q7** | **Q8** | **Q9** | **Q10** | **Q11** | **Q12** | **Q13** | **Q14** |
| --- | --- | --- | --- | --- | --- | --- | --- | --- | --- | --- | --- | --- | --- | --- |
| **Gender** | | | | | | | | | | | | | | |
| Male (father) | 5.1% | 1.6% | 0.0% | 0.8% | 1.2% | 1.2% | 3.5% | 2.4% | 3.9% | 3.9% | 0.8% | 4.3% | 2.0% | 2.8% |
| Female (mother) | 88.6% | 32.7% | 14.2% | 10.2% | 23.2% | 30.3% | 53.9% | 24.0% | 65.0% | 47.2% | 17.3% | 79.1% | 44.1% | 45.3% |
| *p-value* | p>0.05 | p>0.05 | p>0.05 | p>0.05 | p>0.05 | p>0.05 | p>0.05 | p>0.05 | p>0.05 | p>0.05 | p>0.05 | p>0.05 | p>0.05 | p>0.05 |
| **Age** | | | | | | | | | | | | | | |
| 21-30 | 33.9% | 8.7% | 2.8% | 2.0% | 5.1% | 8.3% | 20.1% | 6.7% | 23.2% | 17.7% | 8.3% | 29.5% | 13.4% | 15.4% |
| 31-40 | 41.3% | 17.3% | 8.3% | 5.9% | 13.0% | 15.4% | 23.2% | 12.6% | 29.1% | 20.5% | 5.9% | 37.0% | 21.3% | 21.3% |
| 41-50 | 11.0% | 3.5% | 1.2% | 2.8% | 3.9% | 4.7% | 8.7% | 2.4% | 9.4% | 7.5% | 1.6% | 10.6% | 7.1% | 6.3% |
| 51-60 | 3.9% | 1.6% | 0.8% | 0.4% | 1.6% | 1.6% | 2.4% | 2.0% | 3.9% | 2.8% | 1.2% | 3.1% | 1.6% | 2.8% |
| 61-70 | 2.8% | 2.4% | 1.2% | 0.0% | 0.8% | 1.6% | 2.4% | 2.0% | 2.4% | 2.0% | 1.2% | 2.4% | 2.4% | 1.6% |
| >70 | 0.8% | 0.8% | 0.0% | 0.0% | 0.0% | 0.0% | 0.8% | 0.8% | 0.8% | 0.8% | 0.0% | 0.8% | 0.4% | 0.8% |
| *p-value* | p>0.05 | **0.003** | p>0.05 | p>0.05 | p>0.05 | p>0.05 | p>0.05 | **0.002** | p>0.05 | p>0.05 | p>0.05 | p>0.05 | p>0.05 | p>0.05 |
| **Parents education level** | | | | | | | | | | | | | | |
| Primary | 2.8% | 0.8% | 0.4% | 0.0% | 0.0% | 0.0% | 2.0% | 0.8% | 2.4% | 2.0% | 0.0% | 2.4% | 0.8% | 2.4% |
| Middle | 28.0% | 6.7% | 4.7% | 1.6% | 5.1% | 7.1% | 16.5% | 5.9% | 18.5% | 16.1% | 6.7% | 23.6% | 10.2% | 15.0% |
| Secondary | 33.1% | 10.2% | 2.8% | 2.0% | 7.5% | 11.8% | 20.1% | 7.9% | 25.6% | 15.7% | 5.9% | 29.1% | 16.9% | 13.0% |
| Diploma | 10.2% | 5.5% | 0.8% | 2.8% | 3.1% | 4.3% | 6.7% | 2.8% | 8.7% | 7.1% | 2.0% | 9.4% | 5.9% | 6.7% |
| Undergraduate | 18.5% | 9.8% | 4.3% | 3.5% | 7.5% | 7.5% | 11.4% | 8.3% | 13.4% | 9.4% | 3.5% | 17.7% | 11.0% | 10.2% |
| Graduate or above | 1.2% | 1.2% | 1.2% | 1.2% | 1.2% | 0.8% | 0.8% | 0.8% | 0.4% | 0.8% | 0.0% | 1.2% | 1.2% | 0.8% |
| *p-value* | p>0.05 | **0.000** | **0.000** | **0.000** | **0.001** | p>0.05 | p>0.05 | **0.022** | p>0.05 | p>0.05 | p>0.05 | p>0.05 | **0.012** | **0.024** |
| **Parents profession** | | | | | | | | | | | | | | |
| Medical | 4.7% | 2.8% | 1.2% | 1.2% | 2.4% | 2.8% | 3.9% | 1.6% | 4.3% | 3.1% | 1.6% | 4.7% | 3.5% | 3.5% |
| Non-medical | 89.0% | 31.5% | 13.0% | 9.8% | 22.0% | 28.7% | 53.5% | 24.8% | 64.6% | 48.0% | 16.5% | 78.7% | 42.5% | 44.5% |
| *p-value* | p>0.05 | p>0.05 | p>0.05 | p>0.05 | **0.034** | **0.040** | p>0.05 | p>0.05 | p>0.05 | p>0.05 | p>0.05 | p>0.05 | **0.039** | p>0.05 |
| **Monthly income (in rupiah)** | | | | | | | | | | | | | | |
| 0-1,500,000 | 39.0% | 10.6% | 4.3% | 2.0% | 7.1% | 11.0% | 26.0% | 9.8% | 29.5% | 23.2% | 6.3% | 35.0% | 15.7% | 21.7% |
| 1,500,000-3,000,000 | 24.0% | 8.3% | 3.1% | 3.1% | 6.3% | 7.9% | 15.0% | 4.3% | 17.7% | 13.0% | 5.9% | 24.0% | 12.2% | 12.6% |
| 3,000,000-4,500,000 | 17.3% | 7.5% | 3.1% | 0.8% | 5.9% | 6.3% | 7.9% | 6.7% | 11.8% | 7.5% | 3.5% | 13.0% | 9.1% | 6.3% |
| >4,500,000 | 13.4% | 7.9% | 3.5% | 5.1% | 5.1% | 6.3% | 8.7% | 5.5% | 9.8% | 7.5% | 2.4% | 11.4% | 9.1% | 7.5% |
| *p-value* | p>0.05 | **0.0002** | p>0.05 | **0.000** | **0.034** | p>0.05 | p>0.05 | **0.011** | p>0.05 | p>0.05 | p>0.05 | p>0.05 | **0.020** | p>0.05 |
| **Number of children** | | | | | | | | | | | | | | |
| 1 | 26.0% | 7.5% | 3.1% | 2.0% | 4.7% | 5.1% | 16.9% | 6.7% | 18.5% | 13.0% | 6.7% | 22.4% | 9.8% | 12.6% |
| >1 | 67.7% | 26.8% | 11.0% | 9.1% | 19.7% | 26.4% | 40.6% | 19.7% | 50.4% | 38.2% | 11.4% | 61.0% | 36.2% | 35.4% |
| *p-value* | p>0.05 | p>0.05 | p>0.05 | p>0.05 | p>0.05 | **0.0005** | p>0.05 | p>0.05 | p>0.05 | p>0.05 | p>0.05 | p>0.05 | **0.041** | p>0.05 |
| **Age of children** | | | | | | | | | | | | | | |
| Infancy: 0-24 months | 29.1% | 9.8% | 3.9% | 1.6% | 6.3% | 9.4% | 17.3% | 6.7% | 19.7% | 13.8% | 6.3% | 26.0% | 12.6% | 13.8% |
| Toddler: 25-36 months | 17.3% | 7.5% | 3.1% | 3.5% | 5.1% | 5.1% | 11.4% | 4.7% | 13.0% | 10.2% | 2.8% | 14.6% | 8.7% | 7.9% |
| Early Childhood: 3-5 years | 25.6% | 9.1% | 3.1% | 3.1% | 6.3% | 7.5% | 13.8% | 7.5% | 18.5% | 13.8% | 5.5% | 23.2% | 13.4% | 13.8% |
| Middle Childhood: 6-10 years | 13.4% | 4.3% | 1.6% | 1.6% | 3.1% | 5.1% | 8.3% | 3.9% | 10.2% | 7.1% | 0.8% | 11.8% | 6.3% | 6.3% |
| Late Childhood: 11 years | 8.3% | 3.5% | 2.4% | 1.2% | 3.5% | 4.3% | 6.7% | 3.5% | 7.5% | 6.3% | 2.8% | 7.9% | 5.1% | 6.3% |
| *p-value* | p>0.05 | p>0.05 | p>0.05 | p>0.05 | p>0.05 | p>0.05 | p>0.05 | p>0.05 | p>0.05 | p>0.05 | p>0.05 | p>0.05 | p>0.05 | p>0.05 |
| **Child gender** | | | | | | | | | | | | | | |
| Male | 38.6% | 12.2% | 4.3% | 4.7% | 8.7% | 10.6% | 22.8% | 10.6% | 27.6% | 21.7% | 8.3% | 34.6% | 17.7% | 20.1% |
| Female | 44.1% | 18.1% | 8.3% | 5.5% | 11.8% | 16.1% | 27.6% | 13.0% | 32.7% | 22.0% | 8.3% | 37.4% | 20.9% | 22.4% |
| Both male and female | 11.0% | 3.9% | 1.6% | 0.8% | 3.9% | 4.7% | 7.1% | 2.8% | 8.7% | 7.5% | 1.6% | 11.4% | 7.5% | 5.5% |
| *p-value* | p>0.05 | p>0.05 | p>0.05 | p>0.05 | p>0.05 | p>0.05 | p>0.05 | p>0.05 | p>0.05 | p>0.05 | p>0.05 | p>0.05 | p>0.05 | p>0.05 |
| **Residence** | | | | | | | | | | | | | | |
| Cisaranten Kulon | 46.9% | 14.6% | 6.7% | 2.0% | 10.2% | 15.4% | 25.6% | 9.8% | 31.1% | 22.4% | 7.1% | 37.4% | 21.3% | 22.4% |
| Cisaranten Endah | 6.7% | 2.8% | 0.4% | 0.8% | 1.6% | 2.0% | 3.1% | 2.8% | 5.1% | 3.1% | 1.6% | 5.9% | 2.8% | 3.9% |
| Cisaranten Bina Harapan | 19.7% | 9.1% | 3.5% | 3.9% | 7.9% | 6.3% | 15.4% | 8.3% | 18.1% | 13.0% | 5.1% | 20.5% | 11.4% | 12.2% |
| Sukamiskin | 20.5% | 7.9% | 3.5% | 4.3% | 4.7% | 7.9% | 13.4% | 5.5% | 14.6% | 12.6% | 4.3% | 19.7% | 10.6% | 9.4% |
| *p-value* | p>0.05 | p>0.05 | p>0.05 | **0.003** | p>0.01 | p>0.05 | **0.040** | **0.025** | **0.016** | p>0.05 | p>0.05 | **0.001** | p>0.05 | p>0.05 |

**Supplementary 2.** Bivariate analysis of parental attitudes toward antibiotic use in children

| **Variable** | **Q1** | **Q2** | **Q3** | **Q4** | **Q5** | **Q6** | **Q7** | **Q8** |
| --- | --- | --- | --- | --- | --- | --- | --- | --- |
| **Gender** | | | | | | | | |
| Male (father) | 2.8% | 2.0% | 2.8% | 3.1% | 3.5% | 2.8% | 3.1% | 3.1% |
| Female (mother) | 63.0% | 39.4% | 66.9% | 74.0% | 76.4% | 27.2% | 69.7% | 76.0% |
| p-value | p>0.05 | p>0.05 | p>0.05 | p>0.05 | p>0.05 | p>0.05 | p>0.05 | p>0.05 |
| **Age** | | | | | | | | |
| 21-30 | 21.7% | 10.6% | 22.0% | 26.0% | 27.6% | 9.1% | 23.6% | 26.4% |
| 31-40 | 30.7% | 20.5% | 33.5% | 36.2% | 36.2% | 15.0% | 34.6% | 37.8% |
| 41-50 | 8.7% | 5.9% | 7.9% | 9.1% | 9.8% | 3.5% | 9.1% | 9.8% |
| 51-60 | 2.4% | 2.4% | 3.1% | 2.8% | 3.1% | 1.2% | 2.8% | 3.1% |
| 61-70 | 1.6% | 1.6% | 2.4% | 2.4% | 2.4% | 0.4% | 2.0% | 1.6% |
| >70 | 0.8% | 0.4% | 0.8% | 0.8% | 0.8% | 0.8% | 0.8% | 0.4% |
| *p-value* | p>0.05 | p>0.05 | p>0.05 | p>0.05 | p>0.05 | p>0.05 | p>0.05 | p>0.05 |
| **Parents education level** | | | | | | | | |
| Primary | 1.6% | 0.8% | 1.6% | 2.0% | 2.4% | 1.6% | 1.6% | 1.2% |
| Middle | 18.5% | 10.6% | 19.7% | 22.4% | 24.0% | 9.4% | 19.7% | 24.0% |
| Secondary | 22.0% | 11.0% | 24.8% | 28.3% | 29.5% | 11.0% | 26.8% | 28.3% |
| Diploma | 8.3% | 5.5% | 7.9% | 7.9% | 7.9% | 3.1% | 8.3% | 7.9% |
| Undergraduate | 14.2% | 12.2% | 14.6% | 15.4% | 15.0% | 4.3% | 15.4% | 16.5% |
| Graduate or above | 1.2% | 1.2% | 1.2% | 1.2% | 1.2% | 0.4% | 1.2% | 1.2% |
| p-value | p>0.05 | **0.000** | p>0.05 | p>0.05 | p>0.05 | p>0.05 | p>0.05 | p>0.05 |
| **Parents profession** | | | | | | | | |
| Medical | 3.5% | 3.5% | 3.9% | 3.9% | 3.9% | 0.8% | 3.9% | 4.3% |
| Non-medical | 62.2% | 37.8% | 65.7% | 73.2% | 76.0% | 29.1% | 68.9% | 74.8% |
| *p-value* | p>0.05 | **0.015** | p>0.05 | p>0.05 | p>0.05 | p>0.05 | p>0.05 | p>0.05 |
| **Monthly income (in million)** | | | | | | | | |
| 0-1,500,000 | 25.6% | 15.4% | 27.2% | 31.9% | 32.3% | 13.0% | 29.1% | 32.3% |
| 1,500,000-3,000,000 | 16.5% | 8.7% | 18.1% | 20.5% | 23.2% | 9.1% | 18.5% | 21.3% |
| 3,000,000-4,500,000 | 11.4% | 9.8% | 12.6% | 12.6% | 13.0% | 4.3% | 13.0% | 13.4% |
| >4,500,000 | 12.2% | 7.5% | 11.8% | 12.2% | 11.4% | 3.5% | 12.2% | 12.2% |
| *p-value* | **0.010** | **0.017** | p>0.05 | p>0.05 | p>0.05 | p>0.05 | p>0.05 | p>0.05 |
| **Number of children** | | | | | | | | |
| 1 | 16.5% | 8.3% | 16.1% | 19.7% | 21.7% | 7.5% | 19.3% | 21.3% |
| >1 | 49.2% | 33.1% | 53.5% | 57.5% | 58.3% | 22.4% | 53.5% | 57.9% |
| *p-value* | p>0.05 | **0.024** | **0.017** | p>0.05 | p>0.05 | p>0.05 | p>0.05 | p>0.05 |
| **Age of children** | | | | | | | | |
| Infancy: 0-24 months | 18.9% | 10.2% | 20.1% | 24.4% | 24.4% | 7.1% | 22.4% | 24.0% |
| Toddler: 25-36 months | 11.4% | 8.3% | 13.8% | 14.6% | 15.4% | 5.1% | 13.8% | 14.2% |
| Early Childhood: 3-5 years | 19.7% | 9.8% | 18.1% | 18.5% | 19.7% | 8.7% | 18.9% | 20.9% |
| Middle Childhood: 6-10 years | 9.8% | 8.3% | 11.0% | 13.0% | 13.4% | 7.1% | 11.4% | 12.6% |
| Late Childhood: 11 years | 5.9% | 4.7% | 6.7% | 6.7% | 7.1% | 2.0% | 6.3% | 7.5% |
| *p-value* | p>0.05 | p>0.05 | p>0.05 | p>0.05 | p>0.05 | p>0.05 | p>0.05 | p>0.05 |
| **Child gender** | | | | | | | | |
| Male | 26.8% | 14.2% | 27.6% | 31.9% | 33.5% | 9.4% | 29.1% | 33.1% |
| Female | 31.9% | 22.4% | 33.9% | 36.2% | 36.2% | 16.9% | 35.4% | 37.0% |
| Both male and female | 7.1% | 4.7% | 8.3% | 9.1% | 10.2% | 3.5% | 8.3% | 9.1% |
| *p-value* | p>0.05 | p>0.05 | p>0.05 | p>0.05 | p>0.05 | p>0.05 | p>0.05 | p>0.05 |
| **Residence** | | | | | | | | |
| Cisaranten Kulon | 32.7% | 18.1% | 34.6% | 37.8% | 39.8% | 14.6% | 33.5% | 38.2% |
| Cisaranten Endah | 4.7% | 2.4% | 4.7% | 5.1% | 4.7% | 3.1% | 5.1% | 5.1% |
| Cisaranten Bina Harapan | 14.6% | 11.8% | 15.4% | 16.5% | 17.3% | 6.7% | 16.1% | 18.1% |
| Sukamiskin | 13.8% | 9.1% | 15.0% | 17.7% | 18.1% | 5.5% | 18.1% | 17.7% |
| *p-value* | p>0.05 | p>0.05 | p>0.05 | p>0.05 | p>0.05 | p>0.05 | p>0.05 | p>0.05 |

**Supplementary 3.** Bivariate analysis of parental experience with antibiotic use in children

| **Variable** | **Q1** | **Q2** | **Q3** | **Q4** | **Q5** | **Q6** | **Q7** | **Q8** | **Q9** | **Q10** | **Q11** | **Q12** |
| --- | --- | --- | --- | --- | --- | --- | --- | --- | --- | --- | --- | --- |
| **Gender** | | | | | | | | | | | | |
| Male (father) | 4.3% | 3.1% | 1.6% | 1.6% | 1.6% | 1.2% | 0.8% | 0.8% | 3.5% | 2.4% | 3.1% | 2.8% |
| Female (mother) | 75.2% | 69.7% | 65.7% | 69.7% | 57.5% | 62.6% | 60.2% | 55.9% | 64.6% | 60.2% | 79.5% | 72.4% |
| *p-value* | p>0.05 | p>0.05 | **0.004** | **0.001** | **0.033** | **0.002** | **0.001** | **0.002** | p>0.05 | p>0.05 | 0.039 | p>0.05 |
| **Age** | | | | | | | | | | | | |
| 21-30 | 27.2% | 25.2% | 22.4% | 23.6% | 19.3% | 19.3% | 19.3% | 18.1% | 25.2% | 22.0% | 28.0% | 25.6% |
| 31-40 | 35.4% | 33.5% | 31.5% | 34.3% | 29.5% | 30.7% | 30.3% | 26.8% | 29.9% | 29.1% | 37.8% | 34.3% |
| 41-50 | 9.8% | 8.7% | 8.3% | 7.5% | 6.3% | 8.3% | 6.3% | 6.7% | 7.9% | 7.5% | 10.6% | 9.8% |
| 51-60 | 3.5% | 3.1% | 2.4% | 2.8% | 1.6% | 3.1% | 2.8% | 2.8% | 2.0% | 2.4% | 3.1% | 3.1% |
| 61-70 | 2.8% | 2.0% | 2.4% | 2.8% | 2.0% | 2.0% | 2.0% | 2.0% | 2.4% | 0.8% | 2.4% | 1.6% |
| >70 | 0.8% | 0.4% | 0.4% | 0.4% | 0.4% | 0.4% | 0.4% | 0.4% | 0.8% | 0.8% | 0.8% | 0.8% |
| *p-value* | p>0.05 | p>0.05 | p>0.05 | p>0.05 | p>0.05 | p>0.05 | p>0.05 | p>0.05 | p>0.05 | p>0.05 | p>0.05 | p>0.05 |
| **Parents education level** | | | | | | | | | | | | |
| Primary | 1.6% | 1.6% | 1.6% | 1.6% | 1.2% | 1.2% | 1.2% | 2.0% | 2.0% | 2.0% | 2.0% | 1.2% |
| Middle | 23.6% | 22.8% | 17.3% | 19.7% | 16.5% | 19.3% | 16.9% | 13.4% | 20.9% | 18.5% | 23.2% | 22.4% |
| Secondary | 29.1% | 24.0% | 25.6% | 26.8% | 19.7% | 20.5% | 19.7% | 21.3% | 23.2% | 21.3% | 31.5% | 27.2% |
| Diploma | 8.3% | 7.5% | 7.5% | 6.7% | 6.3% | 7.1% | 7.9% | 7.1% | 6.7% | 6.7% | 8.3% | 8.7% |
| Undergraduate | 15.7% | 15.7% | 14.2% | 15.4% | 14.2% | 14.6% | 14.2% | 11.8% | 14.6% | 13.0% | 16.5% | 14.6% |
| Graduate or above | 1.2% | 1.2% | 1.2% | 1.2% | 1.2% | 1.2% | 1.2% | 1.2% | 0.8% | 1.2% | 1.2 | 1.2% |
| *p-value* | p>0.05 | p>0.05 | p>0.05 | p>0.05 | p>0.05 | p>0.05 | **0.029** | **0.032** | p>0.05 | p>0.05 | p>0.05 | p>0.05 |
| **Parents profession** | | | | | | | | | | | | |
| Medical | 4.7% | 4.3% | 4.3% | 4.7% | 3.5% | 4.3% | 4.3% | 2.8% | 3.5% | 3.1% | 4.7% | 3.5% |
| Non-medical | 74.8% | 68.5% | 63.0% | 66.5% | 55.5% | 59.4% | 56.7% | 53.9% | 64.6% | 59.4% | 78.0% | 71.7% |
| *p-value* | p>0.05 | p>0.05 | p>0.05 | 0.024 | p>0.05 | **0.039** | **0.026** | p>0.05 | p>0.05 | p>0.05 | p>0.05 | p>0.05 |
| **Monthly income (in million)** | | | | | | | | | | | | |
| 0-1.500.000 | 33.9% | 29.5% | 26.0% | 28.0% | 22.0% | 23.6% | 24.0% | 20.9% | 27.6% | 27.6% | 34.6% | 31.5% |
| 1.500.000-3.000.000 | 20.9% | 18.9% | 18.9% | 19.7% | 16.1% | 18.5% | 15.4% | 14.6% | 19.7% | 15.0% | 22.0% | 19.7% |
| 3.000.000-4.500.000 | 12.2% | 12.6% | 11.0% | 12.2% | 10.6% | 11.0% | 10.2% | 9.8% | 11.4% | 9.1% | 13.4% | 12.6% |
| >4.500.000 | 12.6% | 11.8% | 11.4% | 11.4% | 10.2% | 10.6% | 11.4% | 11.4% | 9.4% | 11.0% | 12.6% | 11.4% |
| *p-value* | **0.045** | p>0.05 | p>0.05 | p>0.05 | p>0.05 | p>0.05 | **0.021** | **0.004** | p>0.05 | **0.015** | p>0.05 | p>0.05 |
| **Number of children** | | | | | | | | | | | | |
| 1 | 20.9% | 19.7% | 16.1% | 17.3% | 14.6% | 16.5% | 15.7% | 14.2% | 20.1% | 15.7% | 22.0% | 20.1% |
| >1 | 58.7% | 53.1% | 51.2% | 53.9% | 44.5% | 47.2% | 45.3% | 42.5% | 48.0% | 46.9% | 60.6% | 55.1% |
| *p-value* | p>0.05 | p>0.05 | p>0.05 | p>0.05 | p>0.05 | p>0.05 | p>0.05 | p>0.05 | p>0.05 | p>0.05 | p>0.05 | p>0.05 |
| **Age of children** | | | | | | | | | | | | |
| Infancy: 0-24 months | 21.7% | 21.7% | 18.5% | 20.1% | 17.7% | 18.9% | 17.7% | 17.3% | 21.7% | 17.7% | 24.0% | 21.3% |
| Toddler: 25-36 months | 16.1% | 14.2% | 13.8% | 14.2% | 11.4% | 11.4% | 13.0% | 12.2% | 11.4% | 13.0% | 15.0% | 13.8% |
| Early Childhood: 3-5 years | 21.3% | 19.7% | 17.7% | 18.5% | 17.7% | 18.5% | 17.3% | 14.2% | 18.9% | 19.7% | 22.4% | 20.9% |
| Middle Childhood: 6-10 years | 12.6% | 10.2% | 11.0% | 11.8% | 6.7% | 8.7% | 7.5% | 7.5% | 10.6% | 7.5% | 13.4% | 11.8% |
| Late Childhood: 11 years | 7.9% | 7.1% | 6.3% | 6.7% | 5.5% | 6.3% | 5.5% | 5.5% | 5.5% | 4.7% | 7.9% | 7.5% |
| *p-value* | **0.026** | p>0.05 | p>0.05 | p>0.05 | p>0.05 | p>0.05 | p>0.05 | p>0.05 | p>0.05 | p>0.05 | p>0.05 | p>0.05 |
| **Child gender** | | | | | | | | | | | | |
| Male | 33.9% | 30.3% | 28.0% | 29.5% | 23.2% | 27.2% | 24.8% | 24.0% | 28.3% | 26.8% | 33.5% | 33.1% |
| Female | 36.6% | 34.6% | 32.3% | 33.1% | 28.7% | 29.9% | 29.5% | 25.2% | 31.1% | 29.5% | 38.2% | 33.1% |
| Both male and female | 9.1% | 7.9% | 7.1% | 8.7% | 7.1% | 6.7% | 6.7% | 7.5% | 8.7% | 6.3% | 11.0% | 9.1% |
| *p-value* | p>0.05 | p>0.05 | p>0.05 | p>0.05 | p>0.05 | p>0.05 | p>0.05 | p>0.05 | p>0.05 | p>0.05 | p>0.05 | p>0.05 |
| **Residence** | | | | | | | | | | | | |
| Cisaranten Kulon | 39.8% | 34.3% | 32.3% | 35.0% | 26.8% | 29.9% | 27.2% | 24.8% | 31.1% | 30.3% | 40.9% | 37.4% |
| Cisaranten Endah | 5.1% | 5.1% | 4.3% | 4.3% | 3.9% | 4.7% | 3.9% | 2.8% | 4.3% | 3.5% | 4.7% | 4.3% |
| Cisaranten Bina Harapan | 19.3% | 17.7% | 15.0% | 15.7% | 13.4% | 14.6% | 14.6% | 14.2% | 16.1% | 15.0% | 19.3% | 16.1% |
| Sukamiskin | 15.4% | 15.7% | 15.7% | 16.1% | 15.0% | 14.6% | 15.4% | 15.0% | 16.5% | 13.8% | 17.7% | 17.3% |
| *p-value* | p>0.05 | p>0.05 | p>0.05 | p>0.05 | p>0.05 | p>0.05 | p>0.05 | **0.017** | p>0.05 | p>0.05 | p>0.05 | p>0.05 |
